# Hexahydro-1,3,5-trinitro-1,3,5-triazine (RDX) causes seizure activity in larval zebrafish via antagonism of γ-aminobutyric acid type A receptor α1β2γ2

**DOI:** 10.1007/s00204-023-03475-7

**Published:** 2023-03-13

**Authors:** Paige C. Mundy, Alicia Werner, Latika Singh, Vikrant Singh, Rosalia Mendieta, Caitlyn E. Patullo, Heike Wulff, Pamela J. Lein

**Affiliations:** 1 Department of Molecular Biosciences, Davis School of Veterinary Medicine, University of California, 1089 Veterinary Medicine Drive, Davis, CA 95616 USA; 2 Department of Pharmacology, School of Medicine, University of California, Davis, CA 95616 USA

**Keywords:** GABA_A_R, Positive allosteric modulator (PAM), Royal Demolition Explosive (RDX), Seizures, Zebrafish

## Abstract

Hexahydro-1,3,5-trinitro-1,3,5-triazine, or Royal Demolition Explosive (RDX), is a major component of plastic explosives such as C-4. Acute exposures from intentional or accidental ingestion are a documented clinical concern, especially among young male U.S. service members in the armed forces. When ingested in large enough quantity, RDX causes tonic–clonic seizures. Previous in silico and in vitro experiments predict that RDX causes seizures by inhibiting α1β2γ2 γ-aminobutyric acid type A **(**GABA_A_) receptor-mediated chloride currents. To determine whether this mechanism translates in vivo, we established a larval zebrafish model of RDX-induced seizures. After a 3 h of exposure to 300 µM RDX, larval zebrafish exhibited a significant increase in motility in comparison to vehicle controls. Researchers blinded to experimental group manually scored a 20-min segment of video starting at 3.5 h post-exposure and found significant seizure behavior that correlated with automated seizure scores. Midazolam (MDZ), an nonselective GABA_A_R positive allosteric modulator (PAM), and a combination of Zolpidem (α1 selective PAM) and compound 2-261 (β2/3-selective PAM) were effective in mitigating RDX-triggered behavioral and electrographic seizures. These findings confirm that RDX induces seizure activity via inhibition of the α1β2γ2 GABA_A_R and support the use of GABA_A_R-targeted anti-seizure drugs for the treatment of RDX-induced seizures.

**Supplementary Information:**

The online version contains supplementary material available at 10.1007/s00204-023-03475-7.

## Introduction

Hexahydro-1,3,5-trinitro-1,3,5-triazine, or Royal Demolition Explosive (RDX) is a common component of plasticized explosives, such as C-4, used extensively by militaries worldwide. RDX is also a persistent environmental contaminant found in soil, air, and groundwater surrounding military sites (Gadagbui et al. 2012). It is released via munitions use and from waste generated during its manufacture, packaging, or disposal. RDX has also been detected in indoor air samples of RDX production facilities (Bishop et al. 1988).

Acute exposures to RDX can cause deleterious health outcomes, including severe nausea, vomiting, diarrhea, and, at high doses, generalized seizures (Stone et al. 1969; Kasuske et al. 2009; Whitesides et al. 2021). While accidental occupational ingestion leading to seizures has been reported (Woody et al. 1986; Küçükardali et al. 2003), most acute exposures result from intentional ingestion. During the Vietnam War, it became common knowledge among troops that combined ingestion of small amounts of RDX with alcohol enhanced the effects of ethanol (Stone et al. 1969). Intentional ingestion remains a concern to militaries worldwide, and all published case reports of human ingestion of RDX over the past 20 years have involved military personnel (Whitesides et al. 2021). Of the 31 published case reports from 1969 to 2019, which included accidental and intentional exposures, all involved young male patients, and 100% of the exposed individuals presented with seizures (Whitesides et al. 2021).

The mechanism of RDX-induced seizures was unknown until relatively recently. Based on data obtained using both competitive binding assays and rat brain slice recordings from the amygdala, (Williams et al. 2011), it was suggested that RDX binds to the non-competitive antagonist (NCA) site in the pore of γ-aminobutyric acid type A receptors (GABA_A_R). Recent in silico and in vitro studies from our laboratory corroborated these conclusions (Pressly et al. 2022). Mutations at the so-called “threonine ring” in the pore of the GABA_A_R drastically reduced RDX potency. Further, using whole-cell patch-clamp analyses of recombinantly expressed GABA_A_R, we found that RDX preferentially blocked the GABA_A_R subtype α1β2γ2 in a fully reversible manner (Pressly et al. 2022). However, it remains unknown whether this interaction between RDX and α1β2γ2 GABA_A_R mediates the in vivo seizurogenic activity of RDX.

To address this data gap, we leveraged the larval zebrafish model. Zebrafish express a full range of GABA_A_R subunits, which exhibit an average 80% homology with human GABA_A_Rs (Klee et al. 2012; Monesson-Olson et al. 2018). Larval zebrafish have been proposed as a valid model organism for studying chemical-induced seizures (Griffin et al. 2021), and we and others have used this model to study seizures triggered by GABA_A_R antagonists (Baraban et al. 2005; Bandara et al. 2020). A major advantage of using zebrafish larvae is the medium-to-high-throughput capacity that enables more efficient screening of therapeutics for anti-seizure efficacy (Mundy et al. 2021).

The goals of the current work were to: (1) establish a larval zebrafish model of RDX-induced seizures and (2) leverage this model to determine whether the GABA_A_R subtype selectivity of RDX identified in vitro is recapitulated in vivo as determined using subunit-selective GABA_A_R positive allosteric modulators (PAMs), specifically Zolpidem, an α1-selective PAM (Has et al. 2016), SB-205384, an α6-selective PAM (Heidelberg et al. 2013), and compound 2-261, a β2/3-selective PAM (Sieghart and Savić 2018). The efficacy of these subunit-selective GABA_A_R PAMs in blocking RDX-induced seizures was compared to that of midazolam, a broad-spectrum benzodiazepine considered the standard-of-care treatment for organophosphate-induced seizures (Sigel and Steinmann 2012).

## Methods

### Zebrafish husbandry

Zebrafish husbandry was conducted as previously described in (Mundy et al. 2021). All animal husbandry, spawning and experimental manipulations of zebrafish were approved by the University of California, Davis Institutional Animal Care and Use Committee and conducted in accordance with the ARRIVE guidelines (Percie du Sert et al. 2020). Tropical 5D wild-type zebrafish were obtained from the Sinnhuber Aquatic Research Laboratory (SARL) at Oregon State University, Corvallis, OR, with subsequent generations raised at UC Davis. Adults were housed in a standalone aquatic flow-through system (Aquaneering, San Diego, CA), the water source for which was deionized water from a reverse osmosis system (Reverse Osmosis System Model AAA-1005, Applied Membranes Inc., CA). Standard zebrafish aquaculture conditions (Westerfield and ZFIN 2000) were maintained, including 14:10 h light (~ 850 lx):dark cycle and temperature of 28.5 ± 0.5 °C. A pH of 7.5 ± 0.3 and conductivity of 650 ± 25 μS were maintained via addition of 20 g/L NaHCO_3_ and 40 g/L sea salt solution (Instant Ocean, Blacksburg, VA) to the system water, respectively. Adult fish were fed twice per day with GEMMA Micro 500 (Skretting, Salt Lake City, UT) and placed in false bottom chambers overnight, with males and females separated by a removable divider, to spawn larvae for experiments. In the morning, the divider was removed to permit breeding, and embryos were collected in system water in petri dishes. Embryos were sorted for viability and raised in petri dishes containing embryo media (Westerfield and ZFIN 2000) in a light- and temperature-controlled incubator (14:10 h light:dark) at 29 °C. All experiments were performed on larvae at 5 days post fertilization (dpf).

### Chemical reagents

RDX (CAS: 121-82-4) was obtained from MilliporeSigma (Burlington, MA) as a certified reference material in 1 mg/mL solution in acetonitrile (chromatographic purity 99.9%). For all experiments, the acetonitrile was evaporated and RDX immediately reconstituted in dimethyl sulfoxide (DMSO; CAS: 67-88-5; ≥ 99.7% purity) purchased from Sigma-Aldrich (Saint Louis, MO). Tetramethylenedisulfotetramine (TETS; ≥ 97% purity as determined by GC–MS) was provided by Dr. Bruce Hammock (UC Davis) and synthesized as previously described (Zhao et al. 2014). Midazolam (MDZ) was obtained as a commercially available formulation from Hospira Inc. (Lake Forest, IL) and made as a 5 mg/mL solution in DMSO. Zolpidem (CAS: 82626-48-0; purity ≥ 98% via HPLC) was purchased from Sigma-Aldrich. Compound 2–261 was provided by the laboratory of Kelvin Gee (University of California, Irvine). SB-205384 (CAS: 160296-13-9; purity ≥ 98% via HPLC) was purchased from Tocris (Minneapolis, MN).

Due to limitations in solubility, RDX (and consequently TETS) were prepared as 200 × stocks in DMSO. MDZ, Zolpidem, compound 2–261 and SB-205384 were prepared as 1000 × stocks in DMSO. All stocks were stored at – 20 °C until use. Compounds were diluted to 2×  (initial exposures) or 3×  (treatments following initial exposures) stocks in embryo media just prior to use. The final DMSO concentration for all experiments was below 0.6%.

### Zebrafish exposures and behavior analyses

For all behavioral tests, 4 dpf larval zebrafish were placed in 96-well plates (REF 353,075, Falcon, Irvine, CA) containing 50 µL of embryo media. Parafilm was placed between the top of the plate and the lid to limit evaporation, and plates were placed in an incubator at 29 °C overnight. All behavioral tests were conducted in a DanioVision system (Noldus, Leesburg, VA) with temperature maintained at 28.5 °C and light at 75% (~ 1900 lx). All tests were filmed using a 12 mm lens and video collected at 25 frames per second. For the initial range-finding tests, 5 dpf larvae were exposed to RDX, TETS, or vehicle (DMSO) by adding 50 µL of 2 × media, resulting in final concentrations of either 4 µM TETS (used as a positive control) or 10, 30, 100, or 300 µM RDX. The larvae were filmed for 6 h.

To investigate the efficacy of PAMs in mitigating RDX-induced seizures, 5 dpf larvae were first exposed to 300 µM RDX or vehicle. Parafilm was placed between the top of the plate and lid, and the larvae were placed in a lighted incubator at 29 °C for 3.5 h. After 3.5 h of exposure, larvae were taken out of the incubator and acclimated to the DanioVision system for at least 10 min prior to filming behavior for 20 min. After 20 min of filming, larval zebrafish were exposed to vehicle or varying concentrations of MDZ, Zolpidem, SB-205384, or a combination of 1:1 Zolpidem:compound 2–261 by adding 50 µL of 3 × stock solution of the respective compound in embryo media. The larvae were filmed for 20 min immediately following treatment. All behavioral experiments were repeated at least three times using larvae from separate spawning events with *n* = 16 per treatment per replicate (*n* = 48 total per group).

## Quantification of seizure-like behavior

For the initial tests, EthoVision XT software (Version 15.0; Noldus, Wageningen, the Netherlands) was used to measure total distance moved (mm) in 20-min bins for the entire 6-h exposure window. Each treatment was then compared to each other within each 20-min bin.

Seizure-like behavior was scored both manually and in an automated fashion for a 20-min section of the 6 h initial range-finding test. For manual scoring, researchers blinded to experimental group were provided a 20-min section of the video (03:30–03:50 (hh:mm)) to score using a scoring system similar to previously established zebrafish seizure behavior scoring systems (Baraban et al. 2005; Bandara et al. 2020). Specifically, behavior was scored using the following criteria: Stage I—“hyperlocomotion”, a dramatic increase in swim activity; Stage II—“whirlpool swimming”, rapid “whirlpool-like” circling swim behavior; Stage III—“tonic seizure”, brief clonus-like convulsions leading to loss of posture, e.g., fish falls to one side and remains immobile for 1–3 s.

The same 20-min period that was manually scored (starting at 3.5 h post-exposure) was also analyzed using EthoVision XT. Seizure-like behavior was analyzed as the frequency (total count) of events during which the larvae moved at a velocity greater than 28 mm/s for a duration longer than 1 s. The use of this criterion for seizure-like behavior has been previously described and established as a suitable proxy for manual scoring (Griffin et al. 2021).

## Electrophysiology

Whole-field electrophysiology was conducted as described in (Bandara et al. 2020). Larvae at 5 dpf were immobilized in 300 µM pancuronium bromide (Sigma-Aldrich) in embryo media for 10 min. Next, larvae were mounted in 120 µL 1.5% (w/v) ultraPure^TM^ low-melting agarose (Cat. no. 15517-014, Life Technologies, Carlsbad, CA) made in embryo media. Larvae were mounted on a glass-bottom 35 mm FluoroDish cell culture dish (CAT: FD3510-100, World Precision Instruments, Sarasota, FL) such that the same amount of agarose could be used each time, providing a sturdy and predictable exposure chamber for consistency across treatments. After agarose solidification (~ 5 min), larvae were bathed in 500 µL of either 300 µM RDX or DMSO-only (final concentration 0.5% DMSO) in embryo media. At 3.5 h of exposure, electrophysiological activity was recorded for 10 min. To assess therapeutic ability to mitigate RDX-induced seizures, 500 µL of 2 × stock solution of either DMSO-only, MDZ, Zolpidem, or 1:1 Zolpidem:compound 2-261 were added to the bathing solution at a final concentration of 3 µM MDZ, 10 µM Zolpidem, or 3 µM total 1:1 Zolpidem:compound 2-261, bringing the final concentration of DMSO to 0.6%. Ten minutes after addition of the therapeutics (to allow for proper diffusion into the agar-embedded fish), a 10-min electrophysiological recording was taken.

To record electrical field potentials, a fire-polished glass pipette (micro hematocrit capillary tubes, Cat. no. 22-362-574, Fisherbrand, Waltham, MA), pulled to have a ~ 1 µm opening and backloaded with 2 mM NaCl, was inserted into the optic tectum of the zebrafish larva while at 10× magnification under stereomicroscope. Electrical signal was recorded in a gap-free protocol using A-M Systems model 3000 extracellular amplifier (A-M Systems Inc., Sequim, WA). Voltage records were filtered at low pass 1 kHz, high pass 0.1 Hz, and digitized at 5–10 kHz using a Digidata 1322A analog-to-digital interface (Axon Instruments, Molecular Devices, San Jose, CA). Data were stored using pClamp (Clampex) 9.0 software, and epileptiform activity was analyzed using Clampfit 9.0 software (Molecular Devices). Event thresholds (peak amplitude) were collected as > 3 × baseline with a duration longer than 100 ms. The criteria used for determining seizure events (3 × baseline > 100 ms) has been used previously by our research group to define electrographic seizure events in larval zebrafish, and other research groups have used similar standards (Bandara et al. 2020; Griffin et al. 2021).

### RDX quantification

At 5 dpf, larval zebrafish were exposed to RDX at 300 µM. After a 3.5 h incubation period, 1 mL of exposure water was transferred to a 1.5 mL amber glass screw-top vial and stored at − 20 °C until analysis by high-performance liquid chromatography (HPLC). The larvae were then immobilized temporarily by rapid cooling on an ice bath, depurated three times with fresh embryo media, and then euthanized by placing them in a − 20 °C freezer for 10–20 min.

Euthanized larvae were arranged laterally on Sylgard^™^ (Electron Microscopy Sciences, Hatfield PA). Following a drying period of ~ 8 min, each larva was decapitated using a sterile 16-gauge needle to make an incision extending posterodorsally from the jaw to the base of the hindbrain. Care was taken to not contaminate the heads with yolk from the yolk sac. Heads were pooled in a microcentrifuge tube to collect at least 39 mg of tissue (810 larvae at minimum). Two separate pools of larval heads were collected. For calculation of percent recovery, two microcentrifuge tubes of at least 39 mg of larval head tissue were collected. Larval heads used as DMSO-spiked controls were collected as above without exposure to RDX. Samples were stored at − 80 °C until analysis by HPLC.

To prepare samples for HPLC, heads from RDX-exposed larvae were suspended in 1 mL of acetonitrile with five grinding balls (stainless steel, 2 mm). Heads were crushed using a SPEX SamplePrep Geno/Grinder Homogenizer 2010 (SPEX, Metuchen, NJ) for 2 min at 1500 rpm. The resulting heterogenous mixture was centrifuged for 5 min at 8608× g, and clear supernatant was collected and evaporated to dryness under a constant flow of air using a PIERCE Reacti-Vap^™^ III evaporator (Pierce, IL). The residue was reconstituted in an appropriate volume of a 1:1 mixture of acetonitrile:water. Using this method, the recovery of RDX from larval head samples spiked with 300 μM or 1.5 mM RDX was found to be 100 ± 3%**.**

The certified reference material for the HPLC analysis was RDX (Cerilliant^®^) purchased from MilliporeSigma as a 1.2 mg/1.2 mL solution in acetonitrile (1.2 mL ampules, chromatographic purity 99.9%). RDX concentrations were determined by liquid chromatography using a Hewlett Packard 1100 series HPLC (Palo Alto, CA) equipped with a C-18 Zorbax Eclipse XDB column (5 μm, 4.6 × 150 mm; Agilent, Santa Clara, CA). A commercial RDX stock solution (4.5 mM, 1.2 mg in 1.2 mL acetonitrile) was diluted with a 1:1 mixture of acetonitrile:water to obtain working standards. A 6-point calibration curve ranging from 1 to 1 mM was developed for quantification. The isocratic mobile phase consisted of a 1:1 mixture of acetonitrile:water with a flow rate of 1 mL per minute for 6.0 min at 25 °C. Under these conditions RDX had a retention time of 3.0 min. RDX was monitored by its UV absorption at 234 nm.

### Statistics

For the initial range-finding test, total distance moved for each larva was collected in 20-min bins for the duration of the 6 h static water-borne exposure period. For each 20-min bin, a non-parametric Kruskal–Wallis analysis of variance (ANOVA) was run. For post-hoc testing, Dunn’s multiple comparisons test (*α* = 0.05) was used to compare all treatments to vehicle (DMSO-only) control larvae.

To compare manually *vs*. automatically scored seizure events, the total count of each category of event for the duration of the 20-min filming period was collected. A non-parametric Kruskal–Wallis ANOVA was run, and for post-hoc testing, a Dunn’s multiple comparisons test (*α* = 0.05) was used to compare all treatments to vehicle control (DMSO-only) larvae.

To determine whether automatically scored seizure events were an acceptable proxy for manually scored Stage II events, manual counts for Stage II events in larvae exposed to 100 µM RDX, 300 µM RDX, or 4 µM TETS and their respective automatically scored seizure events were tested in a two-tailed non-parametric Spearman correlation test (*α* = 0.05).

For the EC_50_ analysis, curves were fit to the transformed and normalized behavioral data using nonlinear-least-squares regression with no weighting and Hill slope constrained to – 1. The logEC_50_ values of Zolpidem, 1:1 Zolpidem:compound 2–261, and MDZ were compared using extra sum-of-squares *F* Test (*α* = 0.05).

For the electrophysiology experiments, electrographic bursts were calculated in Clampfit as described in the section "Electrophysiology" and exported to a.txt file. Total bursts for the 10-min epoch were analyzed via non-parametric Kruskal–Wallis test. For the initial validation of electrophysiology activity at 3.5 h of exposure, post hoc testing included a Dunn’s multiple comparisons test (*α* = 0.05) to compare all treatments to vehicle control (DMSO-only) larvae. To test whether PAMs significantly mitigated RDX-induced seizures, post hoc testing included a Dunn’s multiple comparisons test (*α* = 0.05) to compare all treatments to either RDX-only, RDX + MDZ treated or DMSO-only exposed larvae.

Statistical testing including Spearman correlation, EC_50_ generation, and extra sum-of-squares *F* test were conducted in GraphPad Prism (Version 9.2.0; San Diego, CA). All other tests were conducted in R (Version 4.1.1) (R Core Team 2021). Kruskal–Wallis tests were conducted using the package rstatix (Kassambara 2021), and Dunn’s multiple comparisons was completed using the package PMCMRplus (Pohlert 2022). All Dunn’s multiple comparisons included a *p* value adjustment using the Holm-Bonferroni method. Code and example data for R available at https://github.com/insideafish.

## Results

### RDX caused seizure-like behavior and electrographic seizures after 3.5 h of exposure

**Fig. 1:**
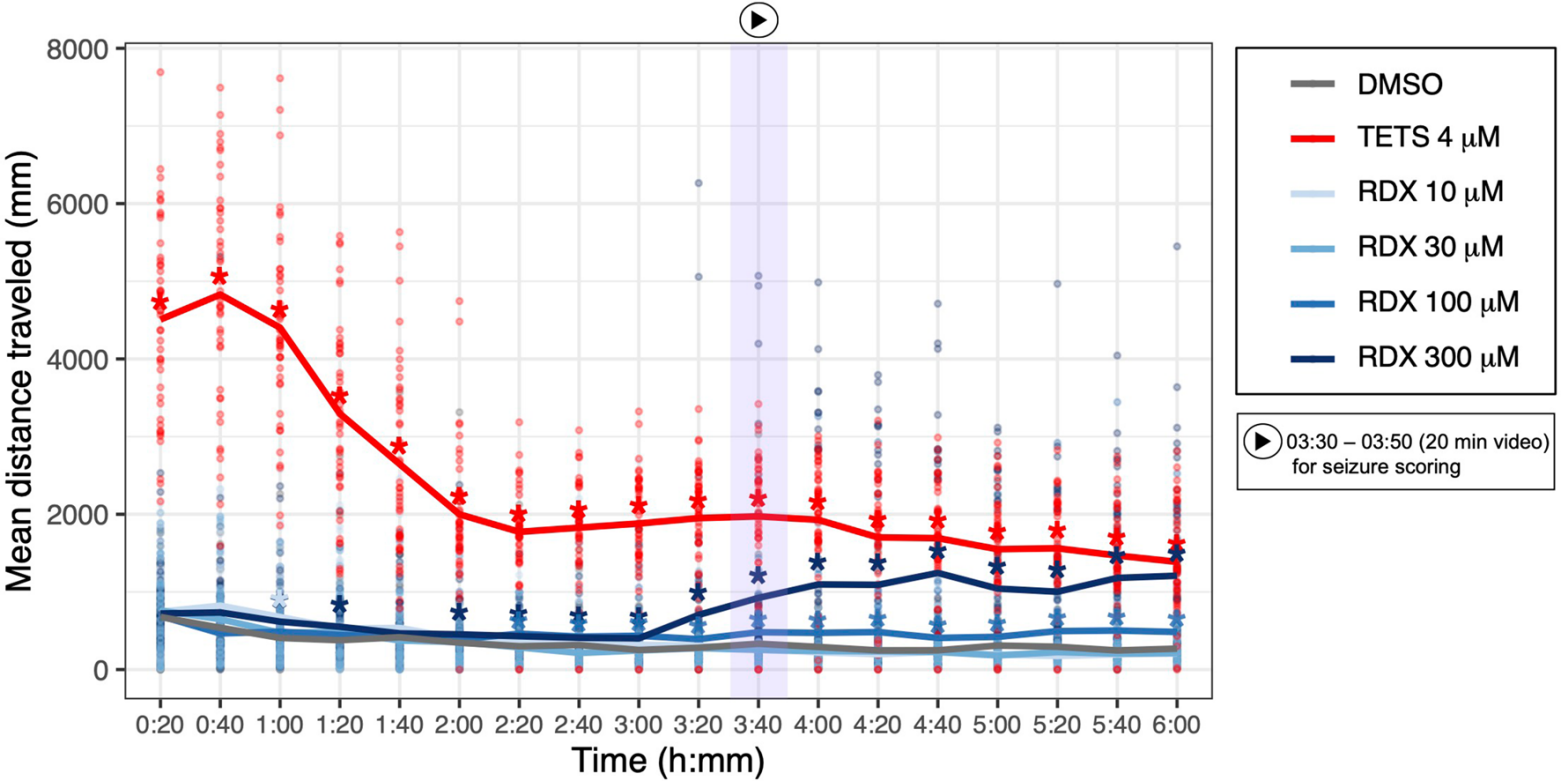
RDX-exposed larval zebrafish exhibited hypermobility after 3.5 h of exposure. Individual dots represent movement of an individual larva. Lines represent the group mean. *Significantly different from DMSO control at *p* < 0.05 in Dunnett’s multiple *t* test (*n* = 46 from at least three individual spawns)

We first aimed to identify the concentration and exposure time at which RDX caused seizures in 5 dpf larval zebrafish because this had not previously been characterized. We exposed 5 dpf larvae to a range of RDX concentrations for 6 h, using 4 µM TETS as a positive control. Total distance moved (mm) was used as a measure of hyperactivity. As expected, larvae exposed to TETS immediately exhibited increased movement (Bandara et al. 2020). In contrast, larvae exposed to 100 µM or 300 µM RDX-only started to exhibit hyperactivity at 2 h of exposure (a 20-min period of 02:00–02:20 hh:mm) (Fig. 1). Larvae exposed to 300 µM of RDX exhibited the greatest increase in hyperactivity after 3 h of exposure, and this level of hypermobility was sustained through to the end of the 6-h exposure (Fig. 1). Larvae exposed to RDX at 10 or 30 µM did not differ significantly from DMSO-only exposed larvae at any time during the 6-h exposure period. We chose to use the 3.5-h exposure time as the start point for quantifying seizures in RDX-exposed larvae since it was the first time point when the mean of total distance moved was at least 2× greater than that of DMSO-only exposed larvae.

**Fig. 2:**
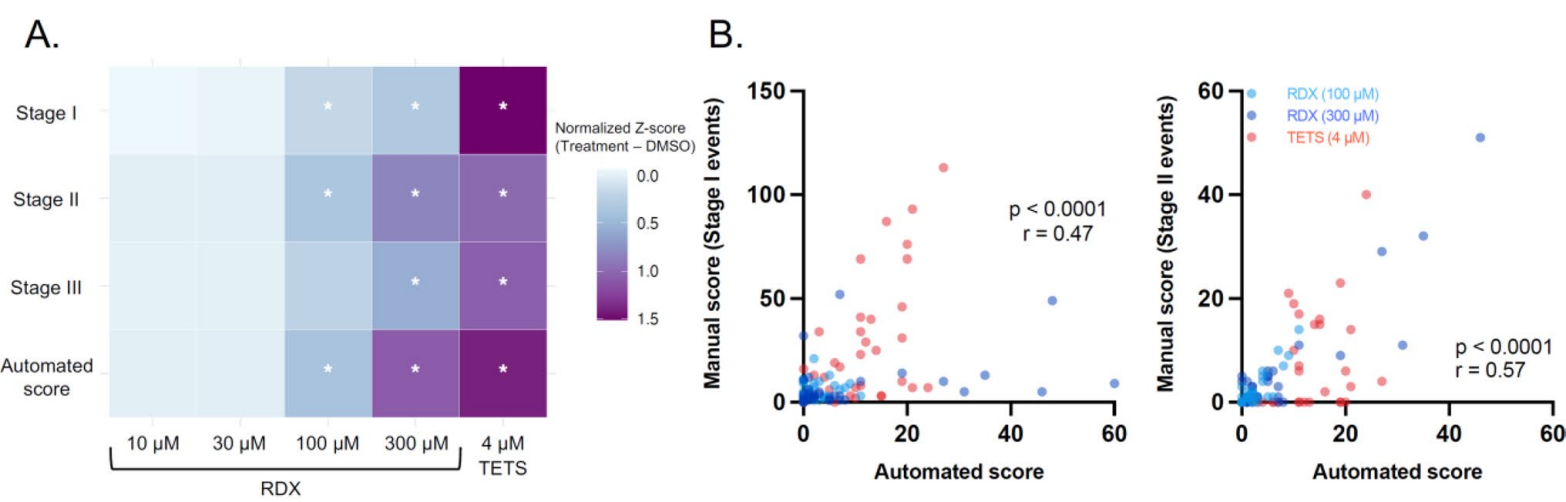
RDX causes seizure-like behavior. **A** Heatmap showing magnitude of directional change between treatment and control groups via normalized *Z*-score (Treatment—DMSO) of manual and automatically scored seizure events. *Significantly different from DMSO-only control at *p* < 0.05, Dunnett’s multiple *t* test (*n* = 36–48 per group, at least three individual spawns). **B** Manual scoring for Stage I and Stage II seizure events and automated scoring for seizure events are significantly correlated. Spearman correlation *α* = 0.05, *n* = 103–105

To investigate whether the RDX-exposed larvae exhibited seizure-like behavior and not simply hyperactivity, researchers blinded to experimental groups scored videos from 03:30 to 03:50 (20 min total) for seizure behavior using previously described methods (Table 1). At this time point, larvae exposed to 100 µM RDX exhibited significantly increased numbers of Stage I and Stage II seizures compared to DMSO-only exposed controls (Fig. 2A). Larvae exposed to 300 µM RDX or 4 µM TETS exhibited significantly increased numbers of Stage I, II, and III seizures. These manual scores were corroborated by automated analyses of the same video clips (Fig. 2A). For graphical purposes only, seizure event counts are presented as a normalized *Z*-score (Treatment – DMSO) to better represent the relative magnitude of directional change of each parameter on the same scale. *Z*-scores were calculated using the following equation: *Z* = (*x* − *μ*)/*σ*, where *x* is the value, *μ* is the mean, and *σ* is the standard deviation. Visual representation of the data for each larva is provided in the supplementary material (Figure S1).

**Table 1:** Criteria used for scoring seizure-like behavior in zebrafish

Collection method	Scored event	Behavior description in zebrafish
Manual	Stage I	“Hyperlocomotion”. Dramatic increase in swim activity
	Stage II	“Whirlpool swimming”. Rapid “whirlpool-like” circling swim behavior
	Stage III	“Tonic seizure”. Brief clonus-like convulsions, leading to loss of posture, e.g., fish falls to one side and remains immobile for 1–3 s
EthoVision XT (Version 15)	Automated	Movement > 28 mm/s for a duration > 1 s

To create a model of RDX-induced seizures that could be used in a medium-to-high-throughput fashion, it would be advantageous to be able to automatically score seizure events. When compared using a Spearman correlation, manually scored Stage II events (for 100 µM RDX, 300 µM RDX, and TETS) significantly correlated with automated seizure scores (Fig. 2B). To ensure no one experimental group was driving the correlation between manual and automated seizure analyses, we analyzed the groups separately. For both Stage I and Stage II events, with the exception of Stage I seizures in the 100 µM RDX group, significant correlations between automated and manual scoring methods were found for all groups (Figure S2).

**Fig. 3:**
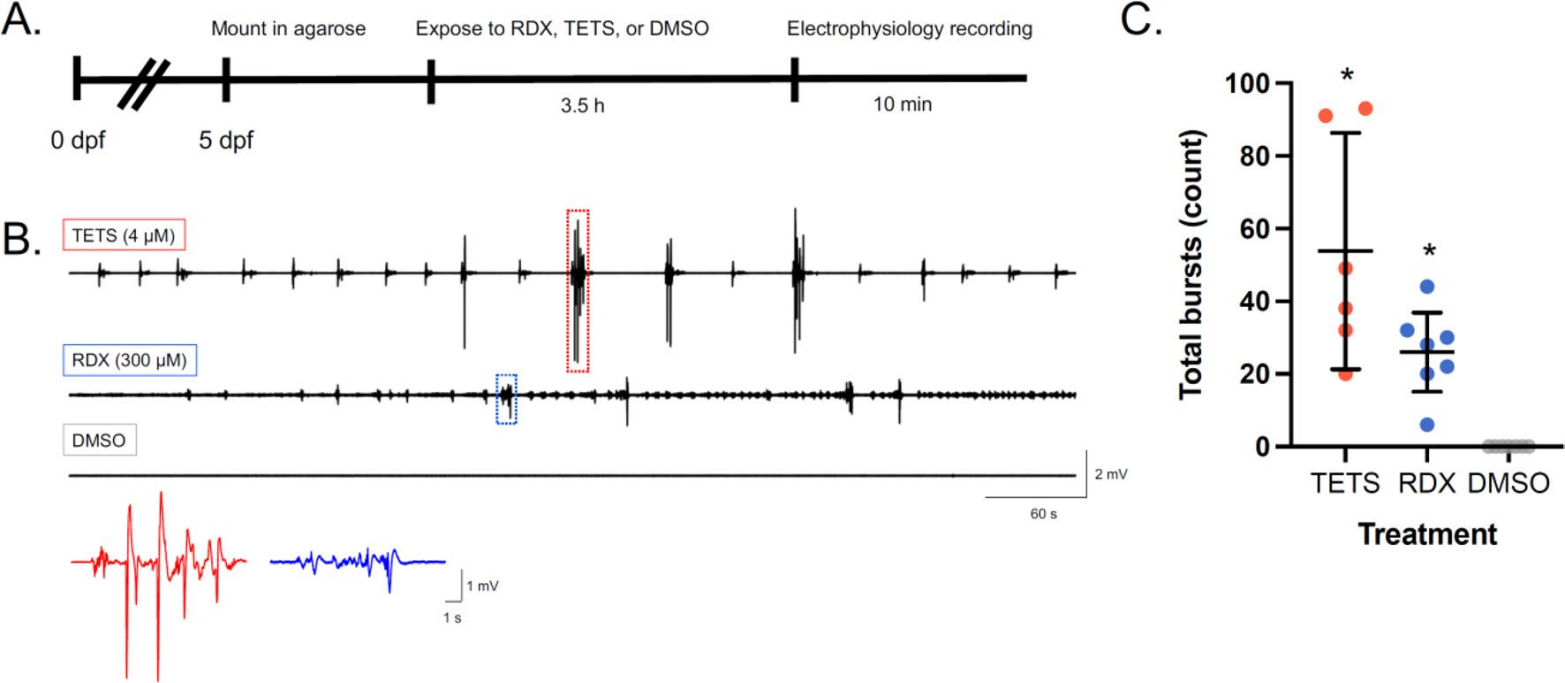
Electrographic seizure activity after 3.5 h of exposure to RDX or TETS. **A** Schematic depicting exposure and data collection paradigm. **B** Representative traces of electrophysiological recordings. Red and blue boxes identify representative multi-spike seizure events, which are shown in corresponding red or blue traces at higher resolution. **C** Total burst counts for the entire 10-min trace. Individual dots represent the sum of bursts for each larva. Bars represent mean ± 95% CI. *p < 0.05 in comparison to DMSO-only in Dunnett’s multiple *t* test (*n* = 6–8 per group, from at least three independent spawns)

To confirm that the seizure-like behaviors we observed corresponded to electrographic seizures, we performed in vivo electrophysiology in larvae exposed to 300 µM RDX, 4 µM TETS, or DMSO for 3.5 h. Larvae were immobilized in low-melting agarose and a signal was recorded from the optic tectum. Both RDX and TETS-exposed larvae experienced a significant number of epileptiform-like electrographic events that were not observed in DMSO-only larvae (Fig. 3).

To confirm that RDX entered zebrafish tissue, we analyzed the amount of RDX uptake into the head tissue of larval zebrafish exposed to 300 µM for 3.5 h. An average concentration of 1324 µM RDX was detected in the tissue sampled, suggesting that RDX concentrates in the heads of exposed larvae (Table 2). Additionally, the average concentration of RDX in exposure water after 3.5 h was found to be 274 µM. The supplementary information (Table S1) provides data on the recovery of RDX from larval head tissue spiked with nominal amounts of RDX.

**Table 2:** RDX concentrations in zebrafish tissue and exposure water

Treatment	Nominal concentration (µM) in exposure water	Number of fish exposed	Weight of sample (pooled heads) (mg)	Measured concentration in heads (µM)	Measured concentration in water (µM)
RDX, replicate 1	300	876	39.6	1401	272
RDX, replicate 2	300	810	39.4	1247	276

### RDX-induced seizures are mitigated by PAMs

**Fig. 4:**
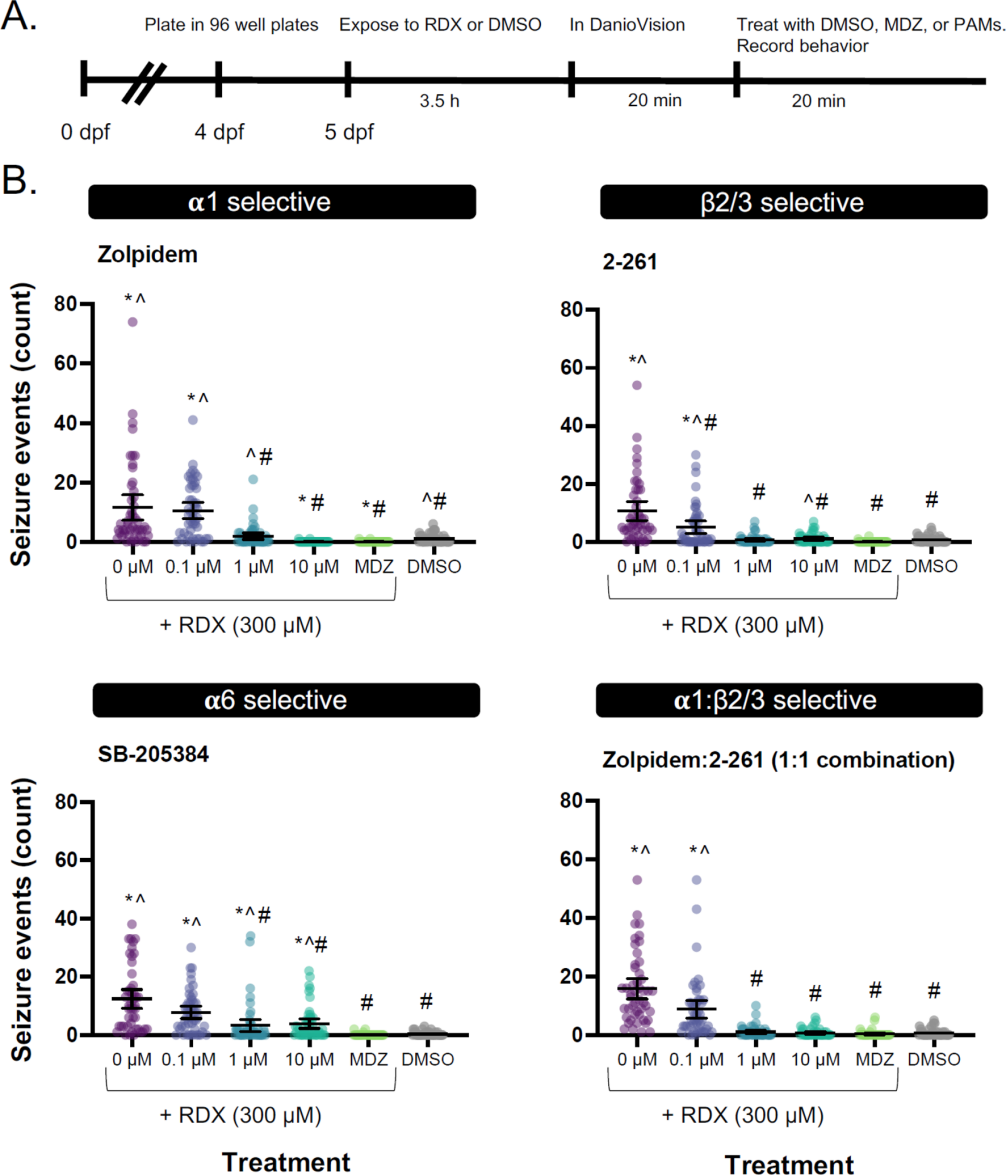
Seizure behavior is mitigated by subunit-selective PAMs. **A** Schematic of the experimental paradigm. **B** Seizure counts in larvae exposed to subunit-selective PAMs after 3.5-h exposure to 300 µM RDX. Individual dots represent individual larvae. Bars are mean ± 95% CI. *p < 0.05 in comparison to DMSO-only, ^*p* < 0.05 in comparison to 3 µM MDZ, #*p* < 0.05 in comparison to RDX-only (0 µM) as determined using Dunnett’s multiple *t* test (*n* = 47–48 larvae from at least three individual spawns)

Once the RDX exposure paradigm had been validated to consistently produce quantifiable seizures, we investigated the anti-seizure efficacy of broad-spectrum and subunit-selective GABA_A_R PAMs in RDX-exposed larval zebrafish. Seizure behavior was automatically quantified in RDX-exposed larvae following treatment with α1-, β2/3-, α6-, or 1:1 combination of α1:β2/3-selective PAMs (Fig. 4). At concentrations of 1 µM, all four treatments demonstrated effectiveness in reducing the number of 300 µM RDX-induced seizure events. Additionally, 10 µM of the α1-selective Zolpidem, 1 µM of the β2/3-selective compound 2–261, or 1 µM of a 1:1 combination of Zolpidem and compound 2–261 decreased seizure activity to approximately the same degree as treatment with 3 µM MDZ. Treatment with the α6-selective PAM SB-205384 was included as a negative control because in vitro studies suggested RDX does not target α6-containing GABA_A_R (Pressly et al. 2022). SB-205384 was unable to decrease seizure activity to the same extent as 3 µM MDZ at any of the concentrations tested.

**Fig. 5:**
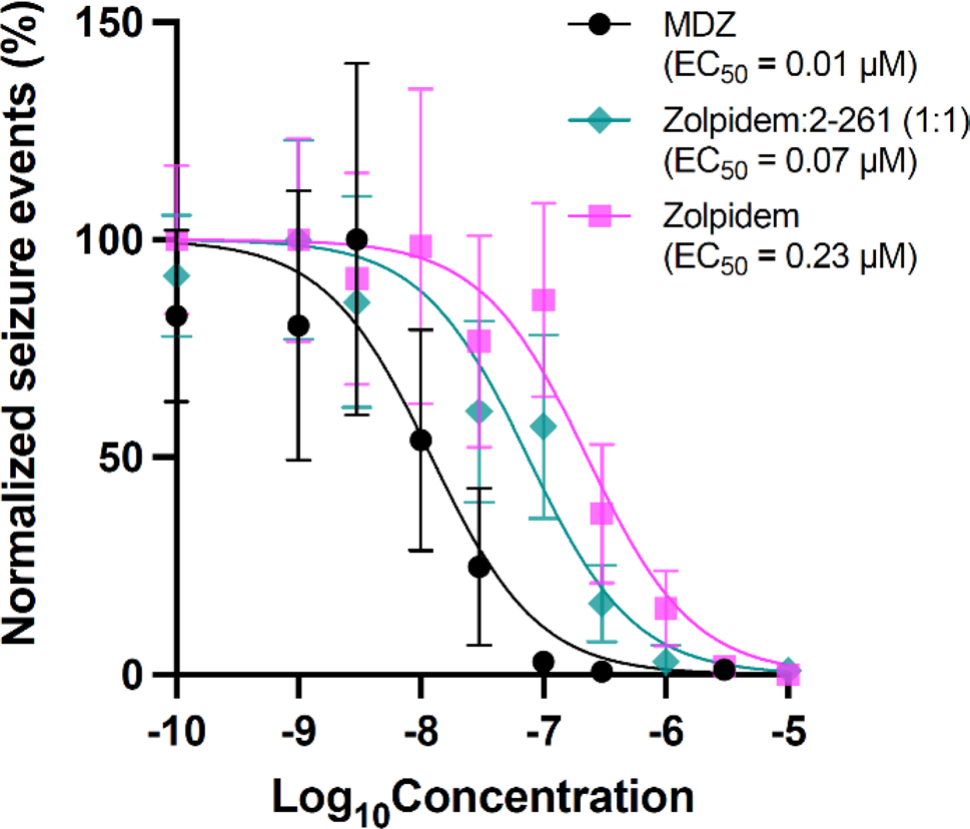
EC_50_ values of individual PAMs differ. Seizure behavior is shown as seizure events (automatically scored) normalized as a percentage within each exposure concentration, with 0% defined as the smallest mean and 100% defined as the largest mean. Individual points represent the mean of normalized seizure count (*n* = 46–144 per group, from at least three independent spawns). Error bars represent mean ± 95% CI. Curves were fit using nonlinear least-squares regression with no weighting and Hill Slope constrained to – 1. The logEC_50_ values between PAMs were compared using extra sum-of-squares *F* test (*α* < 0.05)

A finer concentration range of MDZ, Zolpidem and the 1:1 Zolpidem:compound 2–261 combination was tested to determine EC_50_ values for mitigating RDX-induced seizures. MDZ exhibited an EC_50_ of 0.01 µM; Zolpidem, an EC_50_ of 0.23 µM; and the 1:1 Zolpidem:2–261 combination, an EC_50_ of 0.07 µM (Fig. 5). The EC_50_ values of both Zolpidem and 1:1 Zolpidem:compound 2–261 were significantly different from that of MDZ. Additionally, the EC_50_ values of Zolpidem and 1:1 Zolpidem:compound 2–261 were significantly different from each another.

**Fig. 6:**
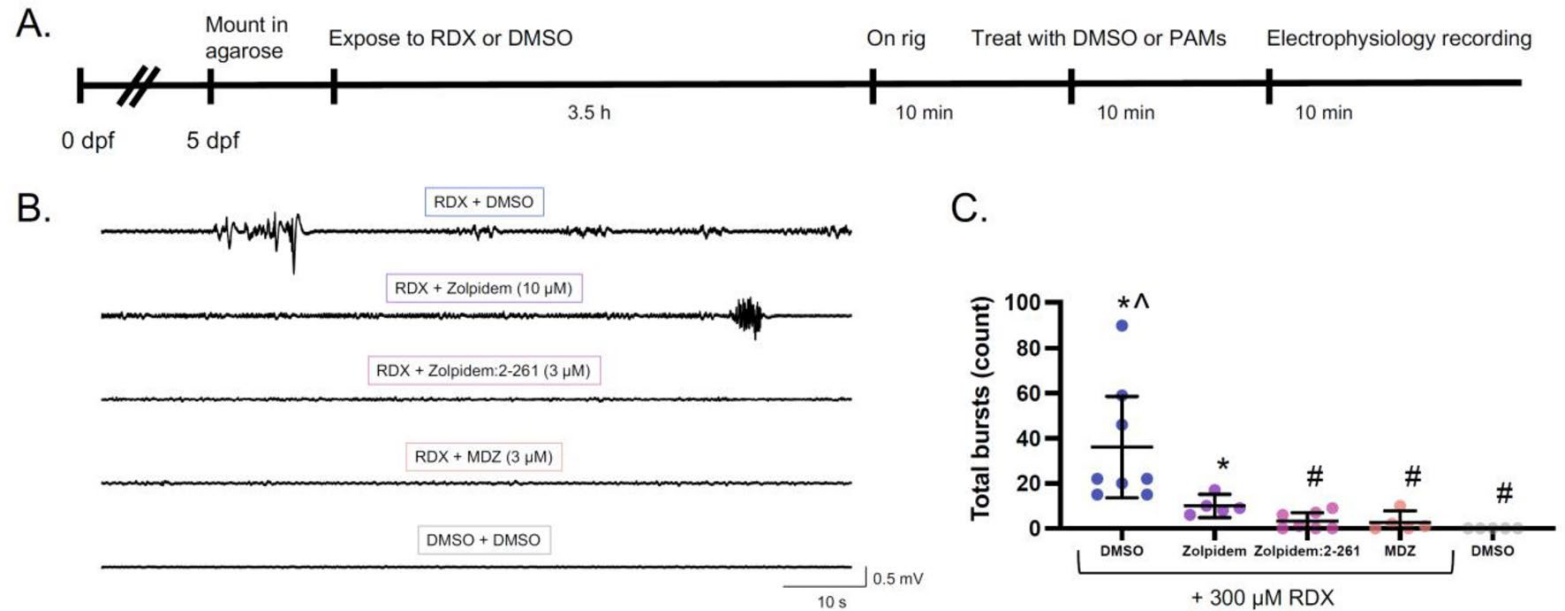
RDX-induced electrographic seizures are reduced by treatment with PAMs. **A** Schematic depicting exposure and data collection paradigm. **B** Representative traces of electrophysiological recordings. **C** Bursting counts within the 10-min trace. Individual dots represent the sum of bursts for each larva. Bars are mean ± 95% CI. *p < 0.05 in comparison to DMSO-only, ^*p* < 0.05 in comparison to MDZ, and #*p* < 0.05 in comparison to RDX-only in Dunnett’s multiple *t* test (*n* = 5–8 larvae per group, from at least three independent spawns)

To investigate whether RDX-induced electrographic seizures were eliminated by treatment with PAMs, we performed in vivo electrophysiology on larvae exposed first to 300 µM RDX for 3.5 h and subsequently treated with PAMs at concentrations that reduced seizure behavior. Treatment with 3 µM MDZ or 1:1 Zolpidem:compound 2–261 significantly reduced the occurrence of electrographic seizure events (Fig. 6). This did not occur with 10 µM Zolpidem.

## Discussion

Seizures induced by ingestion of RDX are a clinical concern worldwide, especially among military personnel. There is currently a lack of consensus regarding the mechanism by which RDX causes seizures. To address this data gap, we developed an in vivo model for RDX-induced seizures using an exposure paradigm that yields consistent and predictable behavioral and electrographic seizures in 5 dpf zebrafish larvae. The 3.5-h delay between the initial exposure and significant seizure activity is consistent with mammalian studies, which report peak RDX concentrations in brain tissue and blood of exposed rats at 3.5 h after exposure (Bannon et al. 2009). We also validated an automated method for scoring seizure events in zebrafish that can be used for medium–high throughput screens. Although total distance moved is often used in zebrafish studies as a proxy for hyperactivity, we sought to use an automated score that aligned more closely with the specific behavior of a tonic–clonic seizure. High-speed, long-duration (> 28 mm/s for > 1 s) events have previously been reported to represent seizure activity in larval zebrafish; further, the same study found that total distance moved was not a reliable measure of seizure activity (Griffin et al. 2021). In this study, manually scored Stage I and II seizure behavior events significantly correlated with automatically scored seizure events. It is likely that the automated events are characteristic of both Stage I and Stage II, considering that velocity and duration, but not direction change, are factored into the automated score (as it would be in a Stage II “cork-screw swimming” behavior).

Both behavioral and electrographic measurements of RDX-induced seizures were less robust than those induced by TETS (used here as a positive control for seizures induced by GABA_A_R antagonist). We hypothesize the difference is due to the fact that TETS targets a different GABA_A_R subunit profile (α2β2γ2) and TETS is roughly 60–100× more potent than RDX. For example, rats intoxicated with 600 µg/kg TETS seized within 5 min of oral administration (Moffett et al. 2019); whereas rats given 75 mg/kg RDX by gavage seized within 11–16 min post-administration (Williams et al. 2011). The length of time necessary for seizures to become evidence in RDX-exposed zebrafish may also reflect in large part the fact that the highest concentration we could test without RDX precipitating out of solution in embryo medium was 300 µM. If it had been possible to test higher RDX concentrations, perhaps we would have observed decreased time to seizure onset and more robust seizures.

MDZ, a broad-spectrum GABA_A_R PAM, is the standard-of-care for treating organophosphate-induced seizures and a first-line anti-seizure medication for treatment of acute seizures (Newmark 2019). Similar to diazepam and lorazepam, MDZ acts on synaptic α1-, α2-, α3- and α5-containing GABA_A_Rs but not on α4- or α6-containing GABA_A_Rs (Olsen and Sieghart 2009; Rudolph and Knoflach 2011). Thus, the observation that MDZ significantly reduced both RDX-induced seizure behavior and electrographic seizure activity in RDX-exposed larval zebrafish provides strong evidence in support of the hypothesis that RDX causes seizures at high concentrations via GABA_A_R antagonism.

Previous in silico and in vitro experiments suggested that RDX preferentially binds to the α1β2γ2 subunit combination. Consistent with this observation, a 1:1 combination of the α1-subunit-selective PAM, Zolpidem, and the β2/3 subunit-selective PAM, compound 2–261, administered at 1 µM or 3 µM was as effective as MDZ at 3 µM in mitigating behavioral and electrographic seizures. However, the EC_50_ of MDZ (0.01 µM) for reducing RDX-induced seizure activity was significantly lower than the 0.07 µM EC_50_ calculated for 1:1 Zolpidem:compound 2-261. This likely reflects the fact that MDZ has activity on a broader profile of GABA_A_R subunits compared to Zolpidem or compound 2–261. However, we cannot rule out the possibility that compared to the subunit-selective PAMs, MDZ reaches higher concentrations in the larval zebrafish brain. The significant difference between EC_50_ values measured for Zolpidem and 1:1 Zolpidem:compound 2-261, with the EC_50_ of the 1:1 combination shifted to the left, suggests that modulation of both subunits is necessary for a more efficient mitigation of seizures. Consistent with the in vitro findings that RDX does not target α6-containing GABA_A_R (Pressly et al. 2022), SB-205384 (an α-6 select PAM) was unable to mitigate seizure-like activity. Combined, these data support the hypothesis that the molecular target of RDX is α1β2γ2. Future studies using site-directed mutagenesis of this receptor in the zebrafish brain will be necessary to confirm this.

Zolpidem, an α1-subunit-selective PAM, significantly decreased seizure-like behavior, but not electrographic seizures, at 10 µM. Zolpidem has sedative properties in mammals and is commonly used as a treatment for insomnia (Monti et al. 2017). This may account for the decreased movement observed in behavioral assays of RDX-exposed zebrafish treated with 10 µM of Zolpidem. Compound 2-261, a β2/3-selective PAM, reduced seizure-like behavior at a lower concentration (0.1 µM) than Zolpidem. The difference in efficacy between compounds could be due to differences in potency or permeability into zebrafish brain tissue. The EC_50_ of compound 2–261 on α1β2γ2 in vitro is reported to be 0.3 µM (Gee et al. 2010), whereas the EC_50_ of Zolpidem on α1β3γ2 in vitro is reported to be 0.48 µM (Has et al. 2016). Further in vivo studies are needed to determine the underlying reason the EC_50_ values differ between these two PAMs.

It is known that modulation of GABA_A_R activity can stop generalized seizures regardless of the primary mechanism of action of the chemoconvulsant. Therapeutically targeting a more abundant subtype such as α1 could have a greater effect than targeting less abundant subtypes. However, we have previously demonstrated that Zolpidem is not effective at mitigating seizures induced by TETS, a GABA_A_R antagonist that selectively targets α2 and α6 subunits (Mundy et al. 2021). Our observations that Zolpidem is effective at mitigating RDX-induced seizures supports our previous in vitro and in silico modeling work (Pressly et al. 2022) that identified the α1 GABA_A_R subunit as a major target of RDX.

Establishing the molecular target of RDX advances our understanding of the mechanisms mediating RDX-induced seizure activity. The in vivo confirmation that the target of RDX, which causes seizure activity, is α1β2γ2 makes physiological sense considering that the α1β2γ2 subunits are estimated to constitute approximately 60% of GABA_A_ receptors and are highly expressed in the cortex, thalamus, pallidum, and hippocampus (Olsen and Sieghart 2009; Rudolph and Knoflach 2011). In several instances of RDX-induced seizures from ingestion, seizures were treated with diazepam (Kasuske et al. 2009), lorazepam (Garcia et al. 2019), or terminated upon reoccurrence with propofol (Garcia et al. 2019; Whitesides et al. 2021). The findings of our study confirm these clinical observations and suggest that effective treatment of RDX-induced seizures should include GABA_A_ modulating drugs such as benzodiazepines, barbiturates, propofol or neurosteroids.

## Supplementary Information

Below is the link to the electronic supplementary material.

Supplementary file1 (DOCX 283 KB)

## Data Availability

The datasets generated during and/or analyzed during the current study are available from the corresponding author on reasonable request.

## Declarations

### Research involving animals

All procedures involving animals were in compliance with the National Institutes of Health Guide for the Care and Use of Laboratory Animals (NIH Publications No. 8253, revised 2011) and ethical approval was granted by the University of California, Davis Institutional Animal and conducted in accordance with the ARRIVE guidelines.
