# Supplementary material for: Hexahydro-1,3,5-trinitro-1,3,5-triazine (RDX) causes seizure activity in larval zebrafish via antagonism of γ-aminobutyric acid type A receptor α1β2γ2
Source: Arch Toxicol. 2023 Mar 13;97(5):1355–65. doi: 10.1007/s00204-023-03475-7 (PMC10110628; doi:10.1007/s00204-023-03475-7)
Supplement: Supplementary file 1 — Supplementary file1 (DOCX 283 KB) [file 204_2023_3475_MOESM1_ESM.docx]

Hexahydro-1,3,5-trinitro-1,3,5-triazine (RDX) causes seizure activity in larval zebrafish via antagonism of γ-aminobutyric acid type A receptor α1β2γ2

SUPPLEMENTARY TABLES

**Table S1.** RDX recovery from larval head tissue.

| **Treatment** | **Nominal concentration (µM)** | **Number of fish exposed** | **Weight of sample (pooled heads) (mg)** | **Measured concentration in heads (µM)** |
| --- | --- | --- | --- | --- |
| Larvae raised in embryo media only | 300 spiked into tissue | 745 | 49.6 | 344 |
| Larvae raised in embryo media only | 1500 spiked into tissue | ~1060 | 54 | 1598 |

SUPPLEMENTARY FIGURES


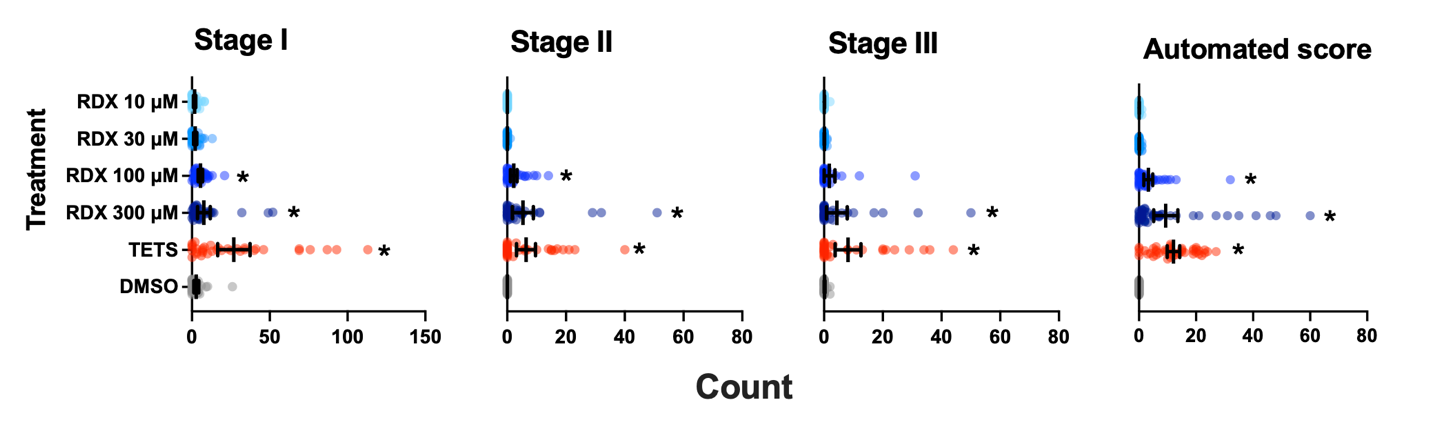


**Figure S1. Manually and automatically counted seizure events at 3.5 h of exposure.** Individual dots represent individual larva (the total count of each score for the entire 20-minute video). Black bars represent mean ± 95% CI. *p<0.05 in Dunns’ multiple comparison test, in comparison to DMSO-only exposed larvae.

**
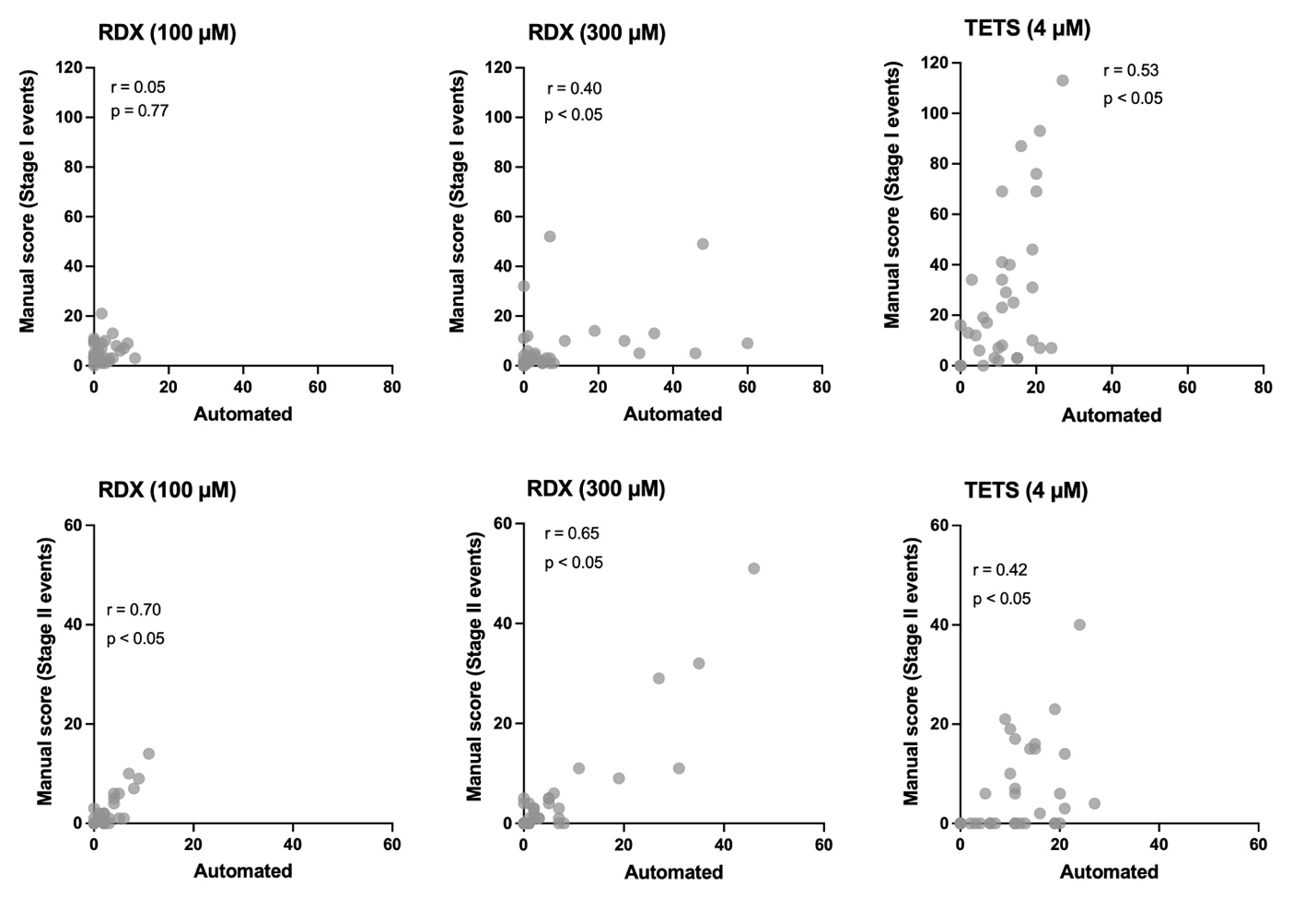
**

**Figure S2. Correlation of manual and automatically scored seizure events for each treatment group.** Spearman correlation where alpha = 0.05. n = 34 – 36 per treatment group.
